# Imbalance in B cell and T Follicular Helper Cell Subsets in Pulmonary Sarcoidosis

**DOI:** 10.1038/s41598-020-57741-0

**Published:** 2020-01-23

**Authors:** I. Kudryavtsev, M. Serebriakova, A. Starshinova, Y. Zinchenko, N. Basantsova, A. Malkova, L. Soprun, L. P. Churilov, E. Toubi, P. Yablonskiy, Y. Shoenfeld

**Affiliations:** 1 St. Petersburg State University, St. Petersburg, Russia; 2 Institute of Experimental Medicine, St. Petersburg, Russia; 3 St. Petersburg Scientific Research Institute of Phthisiopulmonology, St. Petersburg, Russia; 4 Zabludowicz Center for Autoimmune Diseases, Sheba Medical Center, Tel HaShomer, Israel; 5 Sackler Faculty of Medicine, Tel-Aviv University, Tel Aviv, Israel; 6 Faculty of Medicine, Technion, Haifa, Israel; 7 Far Eastern Federal University, Vladivostok, Russia

**Keywords:** Diagnostic markers, Diagnostic markers, Imaging the immune system

## Abstract

Sarcoidosis is a systemic granulomatous disease that develops due to the Th1, Th17 and Treg lymphocytes disturbance. There is an assumption, that B cells and follicular T-helper (Tfh) cells may play an important role in this disorder, as well as in several other autoimmune diseases. The aim of this study was to determine CD19+ B cells subset distribution in the peripheral blood and to define disturbance in the circulating Tfh cells subsets in patients with sarcoidosis. The prospective comparative study was performed in 2016–2018, where peripheral blood B cell subsets and circulating Tfh cell subsets were analyzed in 37 patients with primarily diagnosed sarcoidosis and 35 healthy donors using multicolor flow cytometry. In the results of our study we found the altered distribution of peripheral B cell subsets with a predominance of “naïve” (IgD + CD27−) and activated B cell (Bm2 and Bm2′) subsets and a decreased frequency of memory cell (IgD+ CD27+ and IgD− CD27+) in peripheral blood of sarcoidosis patients was demonstrated. Moreover, we found that in sarcoidosis patients there are increased levels of B cell subsets, which were previously shown to display regulatory capacities (CD24+++ CD38+++ and CD5 + CD27−). Next, a significantly higher proportion of CXCR5-expressing CD45RA − CCR7+ Th cells in patients with sarcoidosis in comparison to the healthy controls was revealed, that represents the expansion of this memory Th cell subset in the disease. This is the first study to demonstrate the association between the development of sarcoidosis and imbalance of circulating Tfh cells, especially CCR4− and CXCR3-expressing Tfh subsets. Finally, based on our data we can assume that B cells and Tfh2- and Tfh17-like cells – most effective cell type in supporting B-cell activity, particularly in antibody production – may be involved in the occurrence and development of sarcoidosis and in several other autoimmune conditions. Therefore, we can consider these results as a new evidence of the autoimmune mechanisms in the sarcoidosis development.

## Introduction

Sarcoidosis is a multisystem granulomatous disease of an unknown etiology. The hallmark of this disease is the presence of non-caseating granulomas that frequently have a pulmonary localization. Granulomas also could be presented in lymph nodes, spleen, eyes, skin, central nervous system or heart. The lung granulomas consist of a variety of immune cells and typically contain the core of macrophages, converted into epithelioid cells and multinucleated giant cells and a shell containing T- and B-cells with the expression of various chemokines and cytokines, including tumor necrosis factor-alpha^1^. Although the exact cause of sarcoidosis remains unknown, a number of etiological factors and pathogenetic pathways have been discussed in the literature. Environmental factors, genetics, foreign (microbial, e.g., mycobacterial or proprionibacterial products and DNA) and self-antigens are considered to play a role in disease development^2^.

Patients with sarcoidosis frequently have higher titers of autoantibodies than healthy subjects, that supports the assumption about the role of autoimmune inflammation in the pathogenesis of the disease^3–5^. Several studies revealed the increased levels of autoantibodies, including antinuclear^3^, anti-dsDNA^5^ or anti-cyclic citrullinated peptide antibodies^4^ in the sera of sarcoidosis patients. Moreover, B cells participate in the specific vimentin recognition that is also support the assumption about the role of humoral immune response in sarcoidosis^6,7^. Next, sarcoidosis is associated with a polyclonal hypergammaglobulinemia, where IgA−, IgG−, and IgM-secreting cells in bronchoalveolar lavage fluid are described^8^. The analysis of IgA and IgG transcripts in them showed high frequencies of somatic hypermutations and increased usage of downstream IgG subclasses, that suggests the prolonged or repetitive immune responses^9,10^. Moreover, the peripheral blood B cell subsets of patients with sarcoidosis were frequently altered in comparison with a healthy controls (НС)^11,12^. Finally, the treatment with anti-B-cell agents (anti-CD20 antibody, Rituximab®) showed a clinical improvement in patients with sarcoidosis suggesting that “naïve” and/or memory B cells may play an important role in this disease^13,14^.

Accordingly, B-cell involvement in sarcoidosis is rather well documented, but still nothing is known about the part of T follicular helper (Tfh) cells in this disease – the major helper T cell subset that considered to be involved in humoral adaptive immune response^15–17^. Tfh cells are essential for the generation of plasma and memory B cells during the germinal center reaction being equipped with different features required for effective B cell help^18,19^. Tfh cells express high levels of the chemokine receptor CXCR5, inducible co-stimulator (ICOS) and CD40 ligand (CD40L) on their cell membrane. CXCR5 is necessary for Tfh migration toward B cell follicles enriched with the CXCL13 while ICOS is a co-stimulatory molecule that is crucial for interactions between Tfh and B cells inside the B cell follicles. In turn CD40L through the interaction with CD40 on the surface of B cell provides signals that are critical for B cell differentiation and class-switching. Additionally, in response to specific stimulation Tfh cells secrete a plenty of IL-21 thereby promoting the growth, differentiation and class-switching of B cells. Recent studies have demonstrated that alterations in circulating Tfh cell subsets have significant effects on the progression of numerous autoimmune diseases^20–23^.

The aim of this study was to determine CD19+ B cells subset distribution in the peripheral blood and to define disturbance in the circulating Tfh cells subsets in patients with sarcoidosis.

## Materials and Methods of the Study

### Маterials of the study

A prospective comparative study was performed in 2016–2018 at the St. Petersburg Scientific Research Institute of Phthisiopulmonology. 37 patients with sarcoidosis stage 2 (men, n = 25 (67.6%), women, n = 12 (32.4%), the average age 32.4 (±6.7) years) and 35 age- and sex-matched control healthy controls (HC) were examined. The clinical characteristics of the studied patients are presented in the Table 1.

**Table 1:** Clinical characteristics of the patients with sarcoidosis.

Characteristics	Pulmonary sarcoidosis, n (%) (n = 37)	CI 95%
**Complaints**		
Clinical manifestations, n (%)	31 (83.8)	0.6848–0.9273
Weakness, n (%)	22 (59.4)	0.4346–0.7368
Cough, n (%)	20 (54.0)	0.3838–0.6897
Chronic fatigue, n (%)	20 (54.0)	0.3838–0.6897
Dyspnea, n (%)	12 (32.4)	0.1955–0.4862
Weight loss, n (%)	8 (21.6)	0.1114–0.3744
Arthralgia, n (%)	8 (21.6)	0.1114–0.3744
Myalgia, n (%)	6 (16.2)	0.0727–0.3152
Sweating, n (%)	2 (5.4)	0.0057–0.1863
Fever, n (%)	11 (2.9)	0.1737–0.4590
**X-ray findings**		
Enlarged lymph nodes	37 (100.0)	0.8880–1.0000
Foci in the lungs, n (%)	34 (91.9)	0.7797–0.9794
Ground-glass opacity, n (%)	7 (18.9)	0.0917–0.3451
Infiltration, n (%)	5 (13.5)	0.0544–0.2845
Fibrosis, n (%)	3 (8.1)	0.0206–0.2203
**Medical history**		
Smoking, n (%)	16 (43.2)	0.2866–0.5910
Family history of autoimmune diseases, n (%)	3 (8.1)	0.0206–0.2203
Allergy, n (%)	16 (4.3)	0.2866–0.5910
**Tests for tuberculosis infection**		
TB.T-SPOT test (positive)	0	0.0000–0.1120
Mantoux test with 2 TE (positive > 5 mm)	7 (18.9)	0.0917–0.3451
Sputum + biopsy specimen microscopy for MBT (positive)	0	0.0000–0.1120
The level of angiotensin converting enzyme (ACE)	14/34 (41.2)	0.2766–0.5241

The diagnosis of pulmonary sarcoidosis was performed according to the standard criteria of the American Thoracic Society (ATS), the European Respiratory Society (ERS) and the World Association of Sarcoidosis and Other Granulomatous Disorders (WASOG). The criteria included typical X-ray changes (mediastinal lymphadenopathy, disseminated foci in lung tissue); histological verification of lung or intrathoracic lymph nodes lesions (detection of epithelioid cell granulomas without caseous necrosis and acid-resistant mycobacteria); exclusion of other causes of granulomatous diseases, primarily tuberculosis.

Exclusion criteria for studied group were: a period of more than 2 years from the evaluation of radiographic changes in the lungs, immunosuppressive and anti-tuberculosis therapy, plasmapheresis for less than 2 months from the date of inclusion, the presence of HIV infection, syphilis, systemic autoimmunity (including undifferentiated connective tissue disease, mixed connective tissue disease, systemic lupus erythematosus (SLE), rheumatoid arthritis (RA), Sjogren’s Syndrome (SS), systemic sclerosis and autoimmune thyroiditis), immunodeficiency, neoplastic diseases and decompensated diabetes mellitus. The inclusion criteria for healthy subjects were: absence of acute and chronic diseases and negative results of immunological tests for tuberculosis infection.

### Methods of the study

All patients underwent a complex examination, including medical survey, multispiral chest computed tomography (MSCT), laboratory blood tests, the level of angiotensin converting enzyme (ACE), standard tuberculosis tests (TB.T-SPOT), histological verification of the lung and intrathoracic lymph nodes lesions (using a transbronchial and videothoracoscopic biopsy).

Blood samples from thirty-seven patients with sarcoidosis and thirty-five age-and sex-matched control healthy subjects were included in this study.

### Peripheral blood B cells immunophenotyping

200 μL of whole peripheral blood were stained on surface with the following specific fluorochrome-conjugated monoclonal antibodies: anti-IgD Alexa Fluor 488 (clone IA6–2, isotype - Mouse IgG2a, κ), anti-CD38 PE (clone LS198-4-3, isotype - IgG1 Mouse), anti-CD183 (CXCR3) PE/Dazzle™ 594 (clone G025H7, isotype - Mouse IgG1, κ), anti-CD27 PC7 (clone 1A4CD27, isotype - IgG1 Mouse), anti-CD24 APC (clone J3–119, isotype - IgG1 Mouse), anti-CD19 APC/Cy7 (clone HIB19, isotype – Mouse IgG1, κ), anti-CD5 Pacific Blue (clone BL1a, isotype - IgG2a Mouse) and anti-CD45 Krome Orange (clone J33, isotype - IgG1 Mouse). IgD, CXCR3 and CD19 were from BioLegend, Inc. (USA), while CD38, CD27, CD24, CD5 and CD45 were from Beckman Coulter, Inc (USA). After incubation at room temperature in the dark for 10 min, erythrocytes were lysed for 15 min with 2 ml of VersaLyse Lysing Solution (Beckman Coulter, Inc., USA) supplied with 50 μL IOTest 3 Fixative Solution (Beckman Coulter, Inc., USA). Next, cells were washed (7 min 330 g) twice with a buffer (sterile phosphate-buffered saline (PBS) containing 2% of heat inactivated fetal bovine serum, Sigma-Aldrich, USA) and were resuspended in 0.5 ml of the PBS containing 2% of neutral buffered formalin solution (Sigma-Aldrich, USA). Sample acquisition was performed using a Navios flow cytometer (Beckman Coulter, Inc., USA), equipped with 405, 488 and 638 nm lasers. At least 5000 CD19+ B cells were analyzed in each sample. Obtained data were analyzed with Kaluza software (Beckman Coulter, Inc., USA). An overview of the gating strategy of the flow cytometric markers can be found in Supplementary Fig. 1.

### Peripheral blood circulating Tfh cells immunophenotyping

200 μL of whole peripheral blood were stained on surface with the following specific fluorochrome-conjugated monoclonal antibodies: anti-CD183 (CXCR3) Alexa Fluor 488 (clone G025H7, iIsotype - Mouse IgG1, k), anti-CD25 PE (clone B1.49.9, isotype - IgG2a Mouse), anti-CD185 (CXCR5) PE/Dazzle™ 594 (clone J252D4, Isotype - Mouse IgG1, k), anti-CD194 (CCR4) PerCP/Cy5.5 (clone L291H4, isotype - Mouse IgG1, k), anti-CD196 (CCR6) PE/Cy7 (clone G034E3, isotype - Mouse IgG2b, κ), anti-CD4 APC (clone 13B8.2, isotype - IgG1 Mouse), anti-CD8 APC-AF700 (clone B9.11, isotype - IgG1 Mouse), anti-CD3 APC/Cy7 (clone HIT3a, isotype - Mouse IgG2a, κ), anti-CD197 (CCR7) Brilliant Violet 421 (clone G043H7, isotype - Mouse IgG2a, κ) and anti-CD45RA Brilliant Violet 510 (clone HI100, Isotype - Mouse IgG2b, κ). CD25, CD4 and CD8 were from Beckman Coulter, Inc (USA), while all other higher-mentioned antibodies – from BioLegend, Inc. (USA). Next, the all samples were processed and analyzed as it was described in earlier in 2.3. At least 40000 CD3 + CD4+ Th cells were analyzed in each sample. An overview of the gating strategy of the flow cytometric markers can be found in Supplementary Fig. 2.

### Statistical analysis

The obtained data were tested for normality of distribution using the Shapiro–Wilk test (all groups contained less than 50 patients). The statistical comparisons of data between sarcoidosis patients and HC were performed using the Mann–Whitney U test. Correlations were assessed using Spearman’s rank correlation coefficient. The differences between the groups were considered significant when p values were <0.05. All of the statistical analysis of data was performed with STATISTICA Version 8.0 Inc. (USA) and GraphPad Prism Version 5.0 (USA).

### Compliance with ethical standards

The study was approved by the Independent Ethical Committee of the St. Petersburg Research Institute of Phthisiopulmonology (protocol No. 34.2 dated 01/19/2017) and the Local Ethical Committee of St. Petersburg State University (protocol No. 01-126 30.06.17). All methods in the study that were confirmed by the Independent Ethical Committee were performed in accordance with the relevant guidelines and regulations (Acceptance Cert №37.9 dated 12/29/2018).

### Informed consent

Informed consent was obtained from all individual participants included in the study.

## Results of the Study

### Peripheral blood B cells

To study possible alterations of B-cell subsets, we first determined the relative number of blood CD19+ B cells both in patients and HC. The frequency of B cells in sarcoidosis patients was significantly higher than in HC (14.48% (9.86; 17.29) vs. 11.09% (8.52; 13.28), p = 0.008, data are not shown). Then we evaluated the percentages of circulating B-cell subsets using two major classification schemes based on the relative co-expression of either IgD and CD38 (so-called “Bm1-Bm5” classification, Suppl. Fig. 1f or IgD and CD27^24^.

#### Increased “activated naïve” and Bm2’ but decreased memory blood B cells in sarcoidosis patients

Thus, IgD and CD38 staining was used to identify “virgin naïve” Bm1 cell IgD + CD38−, “activated naïve” Bm2 cells (IgD + CD38+), pre-germinal-center Bm2′ cells (IgD + CD38++), common subset, containing centroblasts and centrocytes (so-called “Bm3 + Bm4” cells, IgD − CD38++), early memory and resting memory cells (eBm5 and Bm5 cells with the following phenotypes – IgD − CD38+ and IgD − CD38−, respectively)^25^. The percentage ratio of B-cell subsets Bm1-Bm5 is presented in Table 2.

**Table 2:** The percentage ratio of B-cell subsets using “Bm1-Bm5” classification in patients with sarcoidosis and healthy control.

B cell subset	Phenotype	Sarcoidosis (SP, n = 37)	Healthy control (HC, n = 35)	Significant differences
Bm1	IgD + CD38−	10.10 (7.45; 11.69)	15.44 (12.44; 17.62)	↓ <0.001
Bm2	IgD + CD38+	65.40 (62.13; 72.13)	56.79 (49.89; 67.71)	↑ 0.001
Bm2'	IgD + CD38++	8.06 (6.14; 11.35)	3.98 (2.68; 6.27)	↑ <0.001
Bm3 + Bm4	IgD − CD38+++	0.59 (0.28; 0.71)	0.41 (0.28; 0.95)	0.800
eBm5	IgD − CD38+	7.25 (5.26; 11.00)	10.65 (7.90; 15.27)	↓ 0.005
Bm5	IgD − CD38−	4.83 (2.92; 6.50)	8.36 (5.61; 14.26)	↓ <0.001

Note: in Tables 2 and 3 up (“↑”) and down (“↓”) arrows show the significant increase or decrease of compared value in sarcoidosis group vs. control group; the quantitative data are represented as median and quartile ranges (Med (Q25; Q75).

**Table 3:** Comparative analysis of the circulating Tfh subsets in patients with sarcoidosis and healthy control.

Tfh subset	Phenotype	Sarcoidosis (n = 37)	Healthy control (n = 35)	Significant differences
CXCR3 − CCR6 − CCR4− **Tfh**	CXCR3 − CCR6 − CCR4−	13.84 (10.57; 17.70)	12.26 (8.98; 15.30)	0.281
**Tfh2**	CXCR3 − CCR6 − CCR4+	6.90 (5.31; 8.65)	4.67 (3.80; 5.50)	↑ <0.001
**Tfh17**	CXCR3 − CCR6 + CCR4−	20.81 (17.15; 26.11)	22.64 (17.52; 27.77)	0.299
**CCR4+ Tfh17**	CXCR3 − CCR6 + CCR4+	14.53 (10.72; 19.73)	10.67 (9.07; 12.86)	↑ <0.001
**Tfh1**	CXCR3 + CCR6 − CCR4−	22.16 (17.20; 26.53)	27.07 (21.22; 32.43)	↓ 0.015
CXCR3 + CCR6 − CCR4+ **Tfh**	CXCR3 + CCR6 − CCR4+	5.27 (3.64; 6.25)	3.73 (3.20; 4.95)	↑ 0.047
**Tfh17.1**	CXCR3 + CCR6 + CCR4−	10.11 (8.35; 14.16)	14.07 (10.68; 20.01)	↓ 0.004
**DP Tfh17**	CXCR3 + CCR6+CCR4+	3.43 (2.41; 4.99)	2.76 (1.75; 3.32)	↑ 0.006

We found that the frequencies of “activated naïve” Bm2 cells and pre-germinal-center Bm2′ cells were significantly increased in sarcoidosis patients compared to HC. Instead, the frequencies of “virgin naïve” Bm1 cell and the two subsets of memory cells were significantly decreased in blood of the patients when compared with the control values.

#### Altered memory B cell subsets in sarcoidosis patients

**Figure 1:**
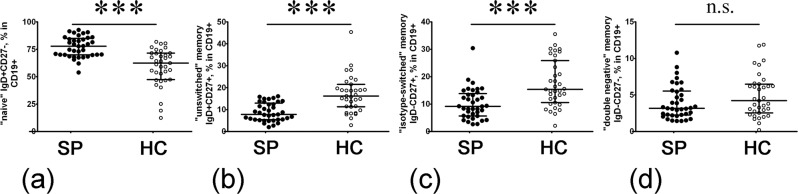
Increased “naïve” but decreased “unswitched” and “isotype-switched” memory peripheral blood B cells in sarcoidosis patients. Scatter plots (**a–d**) showing the percentages “naïve” cells (IgD + CD27−) and three types of memory cells – “unswitched” memory cells (IgD + CD27+), “class-switched” memory cells (IgD − CD27+) and so-called “double-negative” memory cells (IgD − CD27−), respectively, in the peripheral blood samples for sarcoidosis patients (n = 37, black circles, SP) and healthy control subjects (n = 35, white circles, HC). Numbers represent the percentage of the indicated B cell subset among total CD19+ B cell population. Each dot represents individual subjects, and horizontal bars represent the group medians and quartile ranges (Med (Q25; Q75). Statistical analysis was performed with the Mann-Whitney U test (*** – p < 0.001; n.s. – no significance).

Next, within the memory B cell compartment (Suppl. Fig. 1g), we distinguished several subsets. IgD and CD27 staining could distinguish between “naïve” cells (IgD + CD27−) and three types of memory cells – “unswitched” memory cells (IgD + CD27+), “class-switched” memory cells (IgD − CD27+) and so-called “double-negative” memory cells (IgD − CD27−). First of all, the percentage of “naïve” B cells was dramatically increased in sarcoidosis patients vs. HC (77.85% (69.87; 84.25) vs. 62.35% (47.39; 71.42), respectively, p < 0.001, Fig. 1a). Then, we found that the frequency of peripheral blood memory IgD + CD27+ and IgD − CD27+ cells in patients with sarcoidosis was reduced (p < 0.001 for both B cell subsets) compared with HC (Fig. 1b,c).

In contrast, the level of “double-negative” memory B cells did not differ significantly between sarcoidosis patients and HC (3.17% (2.28; 5.45) vs. 4.21% (2.55; 6.48), respectively, p = 0.178, Fig. 1d). These data suggest an altered memory B cell homeostasis in patients with sarcoidosis.

#### Alterations in B cell subsets displaying regulatory properties

**Figure 2:**
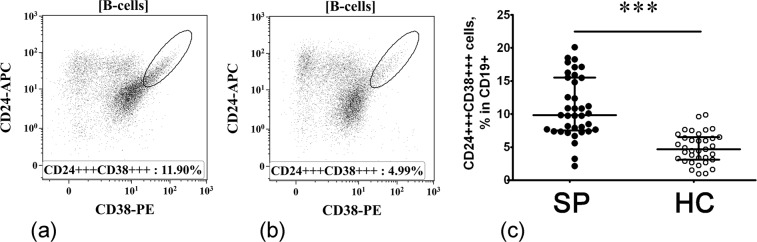
Increased relative number of CD24+++ CD38+++ B cells in patients with sarcoidosis. Representative flow cytometry dot plots of CD24+++ CD38+++ B cell subset among CD19+ B cells in a sarcoidosis patient (**a**) and healthy control subject (**b**). Scatter plots C showing the percentages of CD24+++ CD38++ cell among total B cell population, respectively, in the peripheral blood samples for sarcoidosis patients (n = 37, black circles, SP) and healthy control subjects (n = 35, white circles, HC). Each dot represents individual subjects, and horizontal bars represent the group medians and quartile ranges (Med (Q25; Q75). Statistical analysis was performed with the Mann-Whitney U test. (*** – p < 0.001).

**Figure 3:**
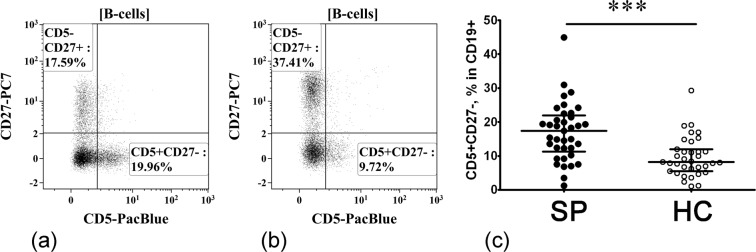
Increased relative number of CD5 + CD27− and decreased number of CD27+ memory B cells in patients with sarcoidosis. Representative flow cytometry dot plots showing expression of CD5 vs. CD27 in a sarcoidosis patient (**a**) and healthy control subject (**b**). Scatter plots C showing the percentages of CD5 + CD27− cell among total B cell population, respectively, in the peripheral blood samples for sarcoidosis patients (n = 37, black circles, SP) and healthy control subjects (n = 35, white circles, HC). Each dot represents individual subjects, and horizontal bars represent the group medians and quartile ranges (Med (Q25; Q75). Statistical analysis was performed with the Mann-Whitney U test. (*** – p < 0.001).

According to the literary data, cell surface phenotype of human Breg includes CD38, CD27, CD24 as well as CD5^7^ and so-called “transitional” CD24+++ CD38++ B cells are considered to be the main IL-10-producing B cells in healthy individuals^26,27^. Primarily, the transitional CD24+++ CD38+++ cells are distinguished within the CD19+ B cell compartment (Fig. 2a) and b). We found that sarcoidosis patients had increased percentages of these cell in their peripheral blood when compared with the HC (9.82% (7.67; 15.48) vs. 4.69% (3.10; 6.52), respectively, p < 0.001, Fig. 2c). Next, we analyzed CD5 versus CD27 expression and distinguished CD5 + CD27− cells within the total B cell population (Fig. 3a,b). Compared with HC, sarcoidosis patients had a significantly increased frequency of CD5 + CD27− cell in circulation (17.41% (12.28; 21.45) vs. 8.21% (5.55; 12.01), respectively, p < 0.001, Fig. 3c). These findings indicate that at least two B cell subsets – CD24+++ CD38+++ and CD5 + CD27−, that according to the literary data were enriched with IL-10-producing cells – are strongly increased in sarcoidosis patients.

Taken together, these results indicate that peripheral blood B cells from patients with sarcoidosis showed an abnormal distribution of “naïve’, memory and regulatory subsets.

### Circulating Tfh

Next, we performed the analysis of Tfh cells that control all the stages of B cell differentiation and activation that are taking place in peripheral lymphoid tissues. It is known that the memory Tfh cells show a “central memory phenotype”, predominantly reside in the secondary lymphoid organs and have the capacity to recirculate in the peripheral blood^28^. Blood memory Tfh cells express short form of CD45RA and CCR7 – cell-surface antigens that are associated with the central memory Th cells. Primarily we estimated the Th distribution on CD45RA and CCR7 expression and distinguished the four main stages of Th maturation – “naïve” (CD45RA + CCR7+), central memory (CM, CD45RA − CCR7+), effector memory (EM, CD45RA − CCR7–) and “effector memory T cells re-expresses CD45RA” or TEMRA (CD45RA + CCR7–) cells (for more details - Suppl. Fig. 2F). Regarding the percentage of circulating Th subsets at different maturation checkpoints, no significant differences were found either in the immature (“naïve”) or in the memory (CM, EM and TEMRA) Th cells between sarcoidosis patients and HC (data not shown).

#### Sarcoidosis patients revealed increased percentage of CXCR5+ central memory Th cells in peripheral blood

To identify the circulating memory Tfh cell, we screened the expression of CXCR5 on circulating central memory Th cells in patients with sarcoidosis and HC. Flow cytometry analysis revealed a significantly higher proportion (p = 0.001) of CXCR5-expressing CM Th cells in patients with sarcoidosis in comparison with HC (39.7% (34.7; 44.6) vs. 32.3% (27.7; 39.2), respectively, data are not shown), representing the expansion of this memory Th cell subset during the disease. Furthermore, in sarcoidosis patients the percentages of CXCR5+ CM Th cells were positively correlated with the values of Bm1 B cell subset (r = 0.370, p = 0.031, Suppl. Fig. 3a) while in the HC this association was statistically insignificant (r = 0.026, p = 0.890, Suppl. Fig. 3b). However, there was no significant correlation between the percentages of CXCR5+ CM Th and CD5 + CD27− B cells in sarcoidosis group (r = 0.010, p = 0.590, data not shown), while in the HC the frequency of Tfh cells positively correlated with CD5 + CD27 − CD19+ lymphocytes (r = 0.467, p = 0.009, data not shown).

#### Imbalance of Tfh subsets in sarcoidosis patients

It should be noted that the standard panel for Tfh subsets analysis is still absent, but according to the available data three main approaches are used for Tfh subsets identification^28,29^. During our experiments we analyzed the cell-surface expression of the chemokine receptors CXCR3 and CCR6 to identify CXCR3 + CCR6− Tfh1−, CXCR3 − CCR6− Tfh2−, and CXCR3 − CCR6+ Tfh17-like cells within CXCR5+ CM Th as it was suggested by Morita and co-authors^30^ (Suppl. Fig. 2h).

**Figure 4:**
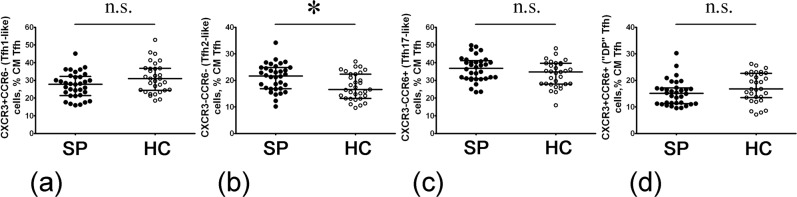
Evaluated level of circulating CXCR3 − CCR6− Tfh2-like cells in sarcoidosis patients peripheral blood. Scatter plots (**a–d**) showing the percentages of CXCR3 + CCR6− Tfh1-like, CXCR3 − CCR6− Tfh2-like, CXCR3 − CCR6+ Tfh17-like and unclassified double-positive CXCR3 + CCR6+ cells among total CD45RA − CCR7+ Tfh population, respectively, in the peripheral blood samples for sarcoidosis patients (n = 37, black circles, SP) and healthy control subjects (n = 35, white circles, HC). Each dot represents individual subjects, and horizontal bars represent the group medians and quartile ranges (Med (Q25; Q75). Statistical analysis was performed with the Mann-Whitney U test. (* – p < 0.05; n.s. – no significance).

Overall analysis of these different Tfh cell subsets in sarcoidosis entailed only CXCR3 − CCR6− Tfh2-like cells which were significantly increased (p = 0.011) when compared with HC (21.7% (16.9; 24.8) vs. 16.6% (13.2; 22.3), respectively, Fig. 4b). However, no significant difference was observed in the distribution of CXCR3 + CCR6− Tfh1−like (p = 0.053) and CXCR3 − CCR6+ Tfh17-like cells (p = 0.127) as well as of unclassified double-positive CXCR3 + CCR6+ cells (p = 0.068).

Interestingly, in the sarcoidosis group a negative correlation was observed between the relative number of Tfh1 cells and CD24+++ CD38+++ B cells (r = –0.387, p = 0.024, see Suppl. Table 1). Furthermore, in HC this relationship was absent, but we observed the positive correlations between the frequencies of Tfh1 cells and Bm2 (r = 0.435, p = 0.016, see Suppl. Table 2) and CD24+++ CD38+++ (r = 0.375, p = 0.041, see Suppl. Table 2), while the frequency of Tfh1 population was negatively correlated not only with the frequency of Bm5, but also with the IgD − CD27+ “switched” memory B cells (r = −0.568, p = 0.001 and r = −0.433, p = 0.017, respectively). However, there were no significant correlations between the percentages of Tfh2 and DP Tfh cells and the frequencies of B cell subsets. But for the relative number of Tfh17 in patients group we found a positive correlation with the percentage of CD24+++ CD38+++ B cells (r = 0.342, p = 0.048). In HC group the relationship between Tfh17 and Bm5 B cells was revealed (r = 0.505, p = 0.004).

Next, we analyzed the CXCR5+ CM Th cell population in patients versus HC, based on chemokine receptors not only CCR6 and CXCR3 co-expression profile but also with CCR4 co-expression (Suppl. Fig. 2i–l). This approach allowed us to specify the phenotype of Tfh1−, Tfh2− and Tfh17-like cells as well as to measure the percentages of several unclassified of Tfh cells (Table 3).

The comparison of these different Tfh subsets between patients and HC indicated significantly higher frequencies of CCR4-expressing Tfh subsets (including Tfh2, CCR4+ Tfh17, DP Tfh17 and unclassified CCR4/CXCR3 co-expressing Tfh) in sarcoidosis patients. However, we did not observe any significant difference in Tfh cells that lacked the expression of all three chemokine receptors as well as in the “classical” Tfh17 that express only CCR6 on their cell membrane. Interestingly, the percentages of two CXCR3-expressing Tfh – CXCR3 + CCR6 − CCR4– (so-called Tfh1) and CXCR3 + CCR6 + CCR4– (so-called Tfh17.1) – were significantly decreased in patients compared with HC (Table 3). These observations indicate that sarcoidosis patients have an abnormal distribution of circulating memory Tfh cell subsets and the imbalance in Tfh cell subsets can be associated with the above-mentioned abnormal distribution of peripheral blood B cell subsets.

Furthermore, we regarded the relationships between the above-mentioned Tfh subsets and different types of B cells (see Suppl. Tables 3 and 4). We found that in sarcoidosis patients the relative number of CXCR3 − CCR6 − CCR4+ Tfh cells negatively correlated with the frequency of “isotype-switched” memory B cells (r = −0.373, p = 0.030). For CXCR3 − CCR6 + CCR4– Tfh cells from sarcoidosis patients peripheral blood the negatively correlated only with Bm3 + Bm4 CD19+ subset (r = −0.485, p = 0.004), while in HC this Tfh subset positively correlated with Bm1 (r = 0.365, p = 0.047), Bm5 (r = 0.534, p = 0.002) and IgD − CD27+ “switched” memory B cells (r = 0.410, p = 0.025). Also, in HC we observed negative relationships between the percentages of CXCR3 − CCR6 + CCR4− Tfh cells and Bm2 (r = −0.440, p = 0.015) and total “naïve” CD5 − CD27− B cell subsets (r = −0.366, p = 0.046). For CXCR3 + CCR6 − CCR4– Tfh cell negatively correlated only with Bm5 IgD − CD38+ B cells (r = −0.501, p = 0.005) in HC. For Tfh17.1 cell we observed positive correlations with “mature” B cell subsets – Bm5 (r = 0.387, p = 0.035) and IgD + CD27+ cells (r = 0.411, p = 0.024) – only in control group. Next, CXCR3 + CCR6 − CCR4+ Tfh cells from peripheral blood of sarcoidosis patients were positively correlated with the relative number of total IgD − CD38+++ Bm3 + Bm4 subset (r = 0.518, p = 0.002). While in HC CXCR3 + CCR6 − CCR4+ Tfh were negatively correlated with Bm1 (r = −0.553, p = 0.002) and IgD + CD27+ (r = −0.539, p = 0.002) B cell subsets. These data suggest that different phenotypes of Tfh cells may have variable functions in regulating the differentiation of B cells during the development of sarcoidosis.

## Discussion

We investigated the frequency and distribution of different B- and T-lymphocyte subsets with the special focus on circulating Tfh cells in sarcoidosis patients compared with HC. We demonstrated that the subset compartment of peripheral blood B cell from patients with pulmonary sarcoidosis was dramatically altered. In line with previous observations, we also indicated the typical disturbances of peripheral B-cell subsets with a decreased frequency of memory B cell subsets and a predominance of “naïve” and activated B cell subsets^11,12^. Remarkably, the pattern of peripheral B cell distribution to different subsets, based on IgD vs. CD38 as well as IgD vs. CD27 co-expression in sarcoidosis patients according to available data was very similar to that reported for SS patients^31,32^.

Our findings revealed, apart from low level of memory B cell, the increased numbers of B cell subsets that were able to display regulatory capacities. Foremost, in peripheral blood samples from sarcoidosis patients we observed higher frequencies of CD24+++ CD38+++ B cells compared with those in matched HC. It was demonstrated that transitional B-cell subsets identified within the CD24highCD38high B cells displayed different regulatory functions including the production of high levels of anti-inflammatory IL-10^33,34^. Moreover, the frequency and absolute number of CD24highCD38high B cells in patients with such inflammatory autoimmune diseases as SS and SLE were significantly higher than in HC. Furthermore, our results agree with previous work showing that high levels of CD24+++ CD38+++ B cells were typical for patients with active sarcoidosis^12^.

Next, we demonstrated the increased level of CD5-expressing B cell in peripheral blood of sarcoidosis patients. Classical papers point to the fact that the CD5+ B cells could be found in various human tissues and were capable of autoantibody production (including rheumatoid factors and anti-ssDNA antibodies). The number of CD5+ B cells is expanded in such autoimmune diseases as RA and SS^33,35^. Unfortunately, little is known about their functional capacities and their precise role in pathophysiologic mechanisms of human autoimmune diseases. From mouse models it is known that CD5-expressing B cell belong to B1a subset that is typically located in the serous cavities and produce low affinity IgM antibodies with autoreactive specificity^36^. In humans CD5 expression could be found on the cell membrane of transitional CD24+++ CD38++ T1 B cells^37^, but according to recent data these cells are able to produce low levels of IL-10 if compared with other transitional B cell subsets^33^.

Considering our current data in the context of these previous reports, we speculate that several Breg subsets could be essentially involved in pathogenesis of sarcoidosis. It should be mentioned that sarcoidosis is a granulomatous disease with focused and targeted migration of the cells presented in granulomata, including lymphoid ones. That’s why any changes in their relative content in peripheral blood may be explained not only by alteration of their production, but also associated with their selective redistribution due to emigration into granulomatous foci. Also, *in vivo*, in course of the disease any changes in the spectrum of peripheral blood cells reflect not only pathogenic but also parallel compensatory processes in the organism, which often causes reciprocal trends. With these pathophysiologic considerations in mind, the data regarding increase in content of Bregs in peripheral blood of sarcoidosis and some other autoimmune patients may reflect either increase in their compensatory production, or pathologic decrease of their migration into foci of inflammation.

Imbalance in B cell subsets could be tightly linked with the abnormal distribution of the Tfh cells that are known to display distinct capacities to help B cells. To our knowledge, so far there were no data concerning peripheral blood Tfh profile related to sarcoidosis patients. We observed that the relative number of CXCR5+ CM Th cell was increased during this disease. It is well-known that impairment of Tfh cell function may underpin some types of autoimmune disorders and several studies registered increased numbers of peripheral blood CD4+ CXCR5+ cells in from patients with SLE^38^, SS^39^, and myasthenia gravis^40^.

Next, we demonstrated that the balance of CD45RA−CCR7+ CXCR5+ Tfh subsets is altered in patients with sarcoidosis. It was found that Tfh2 and Tfh17 cells were able to induce “naïve” B cells to switch isotypes and to promote immunoglobulin secretion (IgG and IgE or IgG and IgA, respectively), while blood memory CXCR3+ Tfh1 cells lacked the capacity to help “naïve” B cells but induced apoptosis in “naïve” activated B cell^30^. Nowadays, the abnormal distribution or imbalance between “regulatory” Tfh1-like cells on one side and “pro-inflammatory” Tfh2 and Tfh17 cells on another side could be closely linked with the pathogenesis of several autoimmune diseases. In our patients we observed an increased percentage of CXCR3 − CCR6− Tfh2-like cells in the circulation which could be consistent with an abnormal humoral immune response. Further analysis of additional Tfh cell subsets co-expressing CCR4, CCR6 and CXCR3 confirmed elevated percentages of CCR4-positive Tfh subsets (including Tfh2, CCR4 + Tfh17, DP Tfh17 and unclassified CCR4/CXCR3 co-expressing Tfh) while CXCR3+ Tfh (Tfh1-like and Tfh17.1-like cells) were decreased.

Unfortunately, no studies have detailed the role of Tfh cell subsets in sarcoidosis, but these cells were intensively studied in main autoimmune disorders. High level of Th2-like and Th17-like CXCR5 + CD4+ T cells was shown in patients with juvenile dermatomyositis^30^, in SS^39^. Moreover, the imbalance in circulating Tfh cell subsets was founded in multiple sclerosis patient’s peripheral blood and it was closely related to the increase of “pro-inflammatory” Tfh17.1 cells and decrease of Th1-like Tfh cells^41,42^. Thus, the frequency of Th2 was significantly higher, while Tfh1 cells were lower in blood samples from patients with active SLE (SLEDAI score > 8) if compared with HC^43^. Furthermore, the alterations in relative numbers of Tfh2 and Tfh1 cells were associated with the presence of autoantibodies in patient’s sera. Next, patients with IgG4-related disease had a significantly increased level of Tfh2 cells compared with HC and Tfh2 relative content was associated with an increased serum IgG4 concentration and the IgG4:IgG ratio in serum^44^. Finally, the increases in circulating Tfh2 and Tfh17 cells were shown in the patients with immunoglobulin A vasculitis compared to HC and, especially noteworthy, the levels of those Tfh subsets positively correlated with serum IgA^45^.

These reports and our current data are highly suggestive that the dominance of Tfh2 and/or Tfh17 subsets over Tfh1 is typical for different systemic and organ-specific autoimmune diseases, as well as for sarcoidosis. Moreover, these observations support the close link between altered Tfh subset balance and pathogenesis of these diseases. Targeting Tfh cells, therefore, appears to be a reasonable strategy for developing new treatments for sarcoidosis and other autoimmune disorders. Recently it was shown that anti-CXCL13 antibody – blocking the main ligand for CXCR5 receptor – demonstrated efficacy at least in two experimental models of autoimmune disorders – the collagen-induced arthritis (a mouse model of RA) and passively-induced experimental autoimmune encephalomyelitis (a Th17-mediated murine model of multiple sclerosis)^46^. Unfortunately, little is known about how the increased “pro-inflammatory” Tfh response proceeded by Tfh2- and Tfh17-like cells is associated with self-reactive B cells. Finally, the revealed changes in B- and T-lymphocyte subsets as well as certain clinical and laboratory similarities between sarcoidosis and a wide range of autoimmune disorders allow us to speculate about the important part of autoimmune components in pathogenesis of sarcoidosis.

The determination of ACE levels is used in the diagnosis of sarcoidosis, however, this biomarker doesn’t have high sensitivity (40–100%) and specificity (83–99%) and can be evaluated in other granulomatous diseases as well^47^. This enzyme is synthesized by lung endothelial cells and converts angiotensin I to angiotensin II. In sarcoidosis the excess of ACE is possibly synthesized by granuloma cells, which causes an increase of this marker in the blood of patients. In our study the level of ACE correlates with the radiological changes and the severity of the disease, and also decreases with immunosuppressive therapy^48^. In this study an increased level of ACE was found in 41% (14/34) of patients, with no correlation with the clinical manifestations of the disease and other biomarkers analyzed. Most likely, this result is associated with a small number of subjects.

## Conclusion

We have demonstrated the altered distribution of peripheral B cell subsets with a decreased frequency of memory B cell subsets and a predominance of “naïve” and activated B cell subsets in sarcoidosis patients. Moreover, we found the increased levels of peripheral blood B cell subsets (CD24+++ CD38+++ and CD5 + CD27−) able to display regulatory capacities. These data point to a potential involvement of B cells in the pathogenesis of sarcoidosis. This is the first study to demonstrate the association of circulating imbalance Tfh cells, especially between CCR4− and CXCR3-expressing Tfh subsets in the development of sarcoidosis.

We found elevated levels of the ACE level in mostly a half of patients, however there is no correlation of these markers with different subtypes of B and T cells. These results might be explained by the small studied group. These new findings raise questions about sarcoidosis pathogenesis and may provide new directions for future clinical studies and treatment strategies.

## Supplementary information

Figure legends.

Supplementary file.

## References

[1] Sakthivel P, Bruder D (2017). Mechanism of granuloma formation in sarcoidosis. Curr. Opin. Hematol..

[2] Miedema JR (2018). Th17-lineage cells in pulmonary sarcoidosis and Löfgren’s syndrome: Friend or foe?. J. Autoimmun..

[3] Kobak S (2014). The prevalence of antinuclear antibodies in patients with sarcoidosis. Autoimmune Dis..

[4] Kobak S, Ylmaz H, Sever F, Duran A, Sen N (2014). Anti-cyclic citrullinated peptide antibodies in patients with sarcoidosis. Sarcoidosis Vasc. Diffuse Lung Dis..

[5] Weinberg I, Vasiliev L, Gotsman I (2000). Anti-dsDNA antibodies in sarcoidosis. Semin Arthritis Rheum..

[6] Eberhardt C (2017). Proteomic Analysis of Kveim Reagent Identifies Targets of Cellular Immunity in Sarcoidosis. PLoS One..

[7] Musaelyana A (2018). Vimentin as antigenic target in autoimmunity: A comprehensive review. Autoimmun Rev..

[8] Hunninghake GW, Crystal RG (1981). Mechanisms of hypergammaglobulinemia in pulmonary sarcoidosis. Site of increased antibody production and role of T lymphocytes. J. Clin. Invest..

[9] Kamphuis LS (2013). Perigranuloma localization and abnormal maturation of B cells: emerging key players in sarcoidosis?. Am J. Respir Crit. Care Med..

[10] Fazel SB, Howie SE, Krajewski AS, Lamb D (1992). B lymphocyte accumulations in human pulmonary sarcoidosis. Thorax..

[11] Lee NS (2011). Disturbed homeostasis and multiple signaling defects in the peripheral blood B-cell compartment of patients with severe chronic sarcoidosis. Clin. Vaccine Immunol..

[12] Saussine A (2012). Active chronic sarcoidosis is characterized by increased transitional blood B cells, increased IL-10-producing regulatory B cells and high BAFF levels. PLoS One..

[13] Belkhou A, Younsi R, Bouchti I, Hassani S (2008). Rituximab as a treatment alternative in sarcoidosis. Joint Bone Spine..

[14] Bomprezzi R, Pati S, Chansakul C, Vollmer T (2010). A case of neurosarcoidosis successfully treated with rituximab. Neurology..

[15] Rao DA (2018). T Cells That Help B Cells in Chronically Inflamed Tissues. Front. Immunol..

[16] Wu H (2018). Molecular Control of Follicular Helper T cell Development and Differentiation. Front. Immunol..

[17] Crotty ST (2019). Follicular Helper Cell Biology: A Decade of Discovery and Diseases. Immunity..

[18] Crotty S (2014). T follicular helper cell differentiation, function, and roles in disease. Immunity..

[19] Vinuesa CG, Linterman MA, Yu D, MacLennan IC (2016). Follicular helper T cells. Annu Rev. Immunol..

[20] Gensous N (2018). T Follicular Helper Cells in Autoimmune Disorders. Front. Immunol..

[21] Park H-J (2014). Insights into the role of follicular helper T cells in autoimmunity. Immune Netw..

[22] Ueno H (2016). T follicular helper cells in human autoimmunity. Curr. Opin. Immunol..

[23] Hunninghake GW (1999). ATS/ERS/WASOG statement on sarcoidosis. American Thoracic Society/European Respiratory Society/World Association of Sarcoidosis and other Granulomatous Disorders. Sarcoidosis Vasc. Diffuse Lung Dis..

[24] Sanz I, Wei C, Lee FE, Anolik J (2008). Phenotypic and functional heterogeneity of human memory B cells. Semin Immunol..

[25] Bohnhorst JO, Bjorgan MB, Thoen JE, Natvig JB, Thompson KM (2001). Bm1-Bm5 classification of peripheral blood B cells reveals circulating germinal center founder cells in healthy individuals and disturbance in the B cell subpopulations in patients with primary Sjögren’s syndrome. J. Immunol..

[26] Bouaziz JD, Le Buanec H, Saussine A, Bensussan A, Bagot M (2012). IL-10 producing regulatory B cells in mice and humans: state of the art. Curr. Mol. Med..

[27] Hasan MM (2019). CD24hiCD38hi and CD24hiCD27+ Human Regulatory B Cells Display Common and Distinct Functional Characteristics. J. Immunol..

[28] Chevalier N (2011). CXCR5 expressing human central memory CD4 T cells and their relevance for humoral immune responses. J. Immunol..

[29] Ueno H, Banchereau J, Vinuesa CG (2015). Pathophysiology of T follicular helper cells in humans and mice. Nat. Immunol..

[30] Morita R (2011). Human blood CXCR5(+)CD4(+) T cells are counterparts of T follicular cells and contain specific subsets that differentially support antibody secretion. Immunity..

[31] Binard A (2009). Is the blood B-cell subset profile diagnostic for Sjogren syndrome?. Ann Rheum Dis..

[32] Bohnhorst JO, Thoen JE, Natvig JB, Thompson KM (2001). Significantly depressed percentage of CD27+ (memory) B cells among peripheral blood B cells in patients with primary Sjögren’s syndrome. Scand J. Immunol..

[33] Simon Q (2016). In-depth characterization of CD24(high)CD38(high) transitional human B cells reveals different regulatory profiles. J. Allergy Clin Immunol..

[34] Burastero SE, Casali P, Wilder RL, Notkins AL (1988). Monoreactive high affinity and polyreactive low affinity rheumatoid factors are produced by CD5+ B cells from patients with rheumatoid arthritis. J. Exp. Med..

[35] Dauphinée M, Tovar Z, Talal N (1988). B cells expressing CD5 are increased in Sjögren’s syndrome. Arthritis Rheum..

[36] Kantor AB, Herzenberg LA (1993). Origin of murine B cell lineages. Annu Rev. Immunol..

[37] Sims GP (2005). Identification and characterization of circulating human transitional B cells. Blood..

[38] Simpson N (2010). Expansion of circulating T cells resembling follicular helper T cells is a fixed phenotype that identifies a subset of severe systemic lupus erythematosus. Arthritis Rheum..

[39] Li XY (2012). Role of the frequency of blood CD4(+) CXCR5(+) CCR6(+) T cells in autoimmunity in patients with Sjögren’s syndrome. Biochem Biophys Res. Commun..

[40] Luo C (2013). Expansion of circulating counterparts of follicular helper T cells in patients with myasthenia gravis. J. Neuroimmunol..

[41] Cunill V (2018). Relapsing-Remitting Multiple Sclerosis Is Characterized by a T Follicular Cell Pro-Inflammatory Shift, Reverted by Dimethyl Fumarate Treatment. Front Immunol..

[42] Christensen JR (2013). Systemic inflammation in progressive multiple sclerosis involves follicular T-helper, Th17- and activated B-cells and correlates with progression. PLoS One..

[43] Le Coz C (2013). Circulating TFH subset distribution is strongly affected in lupus patients with an active disease. PLoS One..

[44] Akiyama M (2015). Number of Circulating Follicular Helper 2 T Cells Correlates With IgG4 and Interleukin-4 Levels and Plasmablast Numbers in IgG4-Related Disease. Arthritis Rheumatol..

[45] Liu D (2017). Distribution of circulating T follicular helper cell subsets is altered in immunoglobulin A vasculitis in children. PLoS One..

[46] Klimatcheva E (2015). CXCL13 antibody for the treatment of autoimmune disorders. BMC Immunol..

[47] Yang H, Mo T, Nie W, Li B (2016). Angiotensin converting enzyme I/D polymorphism and sarcoidosis risk. Sarcoidosis Vasc. Diffuse Lung Dis..

[48] Vorselaars AD (2015). ACE and sIL-2R correlate with lung function improvement in sarcoidosis during methotrexate therapy. Respir. Med..

