# Supplementary material for: Imbalance in B cell and T Follicular Helper Cell Subsets in Pulmonary Sarcoidosis
Source: Sci Rep. 2020 Jan 23;10:1059. doi: 10.1038/s41598-020-57741-0 (PMC6978348; doi:10.1038/s41598-020-57741-0)
Supplement: Supplementary file 1 — Figure legends. [file 41598_2020_57741_MOESM1_ESM.doc]

Figure legends.

**Imbalance in B cell and T follicular helper cell subsets in pulmonary sarcoidosis**

Kudryavtsev I.^1,2,7^, Serebriakova M.^1,2^, Starshinova A.^1^, Zinchenko Y.^1,3^, Basantsova N.^1,3^, Malkova A.^1^, Soprun L.^1^, Churilov L.^1,2^ , Toubi E.^6^, Yablonsky P.^1,3^, Shoenfeld Y.^1,4,5^

St. Petersburg State University, St. Petersburg, Russia^1^

Institute of Experimental Medicine, St. Petersburg, Russia^2^

St. Petersburg Scientific Research Institute of Phthisiopulmonology, St. Petersburg, Russia^3^

Zabludowicz Center for Autoimmune Diseases, Sheba Medical Center, Tel HaShomer, Israel ^4^

Sackler Faculty of Medicine, Tel-Aviv University, Tel Aviv, Israel ^5^

Faculty of Medicine, Technion, Haifa, Israel^6^

Far Eastern Federal University, Vladivostok, Russia^7^

**Figure 1.** Increased “naïve” but decreased “unswitched” and “isotype-switched” memory peripheral blood B cells in sarcoidosis patients.

Scatter plots a), b), c) and d) showing the percentages “naïve” cells (IgD+CD27–) and three types of memory cells – “unswitched” memory cells (IgD+CD27+), “class-switched” memory cells (IgD–CD27+) and so-called “double-negative” memory cells (IgD–CD27–), respectively, in the peripheral blood samples for sarcoidosis patients (n=37, black circles, SP) and healthy control subjects (n=35, white circles, HC). Numbers represent the percentage of the indicated B cell subset among total CD19+ B cell population. Each dot represents individual subjects, and horizontal bars represent the group medians and quartile ranges (Med (Q25; Q75). Statistical analysis was performed with the Mann-Whitney U test.

**Figure 2.** Increased relative number of CD24+++CD38+++ B cells in patients with sarcoidosis.

Representative flow cytometry dot plots of CD24+++CD38+++ B cell subset among CD19+ B cells in a sarcoidosis patient (a) and healthy control subject (b). Scatter plots C showing the percentages of CD24+++CD38++ cell among total B cell population, respectively, in the peripheral blood samples for sarcoidosis patients (n=37, black circles, SP) and healthy control subjects (n=35, white circles, HC). Each dot represents individual subjects, and horizontal bars represent the group medians and quartile ranges (Med (Q25; Q75). Statistical analysis was performed with the Mann-Whitney U test.

**Figure 3.** Increased relative number of CD5+CD27– and decreased number of CD27+ memory B cells in patients with sarcoidosis.

Representative flow cytometry dot plots showing expression of CD5 vs. CD27 in a sarcoidosis patient (a) and healthy control subject (b). Scatter plots C showing the percentages of CD5+CD27– cell among total B cell population, respectively, in the peripheral blood samples for sarcoidosis patients (n=37, black circles, SP) and healthy control subjects (n=35, white circles, HC). Each dot represents individual subjects, and horizontal bars represent the group medians and quartile ranges (Med (Q25; Q75). Statistical analysis was performed with the Mann-Whitney U test.

**Figure 4.** Evaluated level of circulating CXCR3−CCR6− Tfh2-like cells in sarcoidosis patients peripheral blood.

Scatter plots a), b), c) and d) showing the percentages of CXCR3+CCR6− Tfh1-like, CXCR3−CCR6− Tfh2-like, CXCR3−CCR6+ Tfh17-like and unclassified double-positive CXCR3+CCR6+ cells among total CD45RA–CCR7+ Tfh population, respectively, in the peripheral blood samples for sarcoidosis patients (n=37, black circles, SP) and healthy control subjects (n=35, white circles, HC). Each dot represents individual subjects, and horizontal bars represent the group medians and quartile ranges (Med (Q25; Q75). Statistical analysis was performed with the Mann-Whitney U test.
