# Supplementary material for: Imbalance in B cell and T Follicular Helper Cell Subsets in Pulmonary Sarcoidosis
Source: Sci Rep. 2020 Jan 23;10:1059. doi: 10.1038/s41598-020-57741-0 (PMC6978348; doi:10.1038/s41598-020-57741-0)
Supplement: Supplementary file 2 — Supplementary file. [file 41598_2020_57741_MOESM2_ESM.doc]

**Imbalance in B cell and T follicular helper cell subsets in pulmonary sarcoidosis**

Kudryavtsev I.^1,2,7^, Serebriakova M.^1,2^, Starshinova A.^1^, Zinchenko Y.^1,3^, Basantsova N.^1,3^, Malkova A.^1^, Soprun L.^1^, Churilov L.^1,2^ , Toubi E.^6^, Yablonsky P.^1,3^, Shoenfeld Y.^1,4,5^

St. Petersburg State University, St. Petersburg, Russia^1^

Institute of Experimental Medicine, St. Petersburg, Russia^2^

St. Petersburg Scientific Research Institute of Phthisiopulmonology, St. Petersburg, Russia^3^

Zabludowicz Center for Autoimmune Diseases, Sheba Medical Center, Tel HaShomer, Israel ^4^

Sackler Faculty of Medicine, Tel-Aviv University, Tel Aviv, Israel ^5^

Faculty of Medicine, Technion, Haifa, Israel^6^

Far Eastern Federal University, Vladivostok, Russia^7^

**Supplementary Figure 1.** B cell flow cytometry immunophenotyping gating strategy.


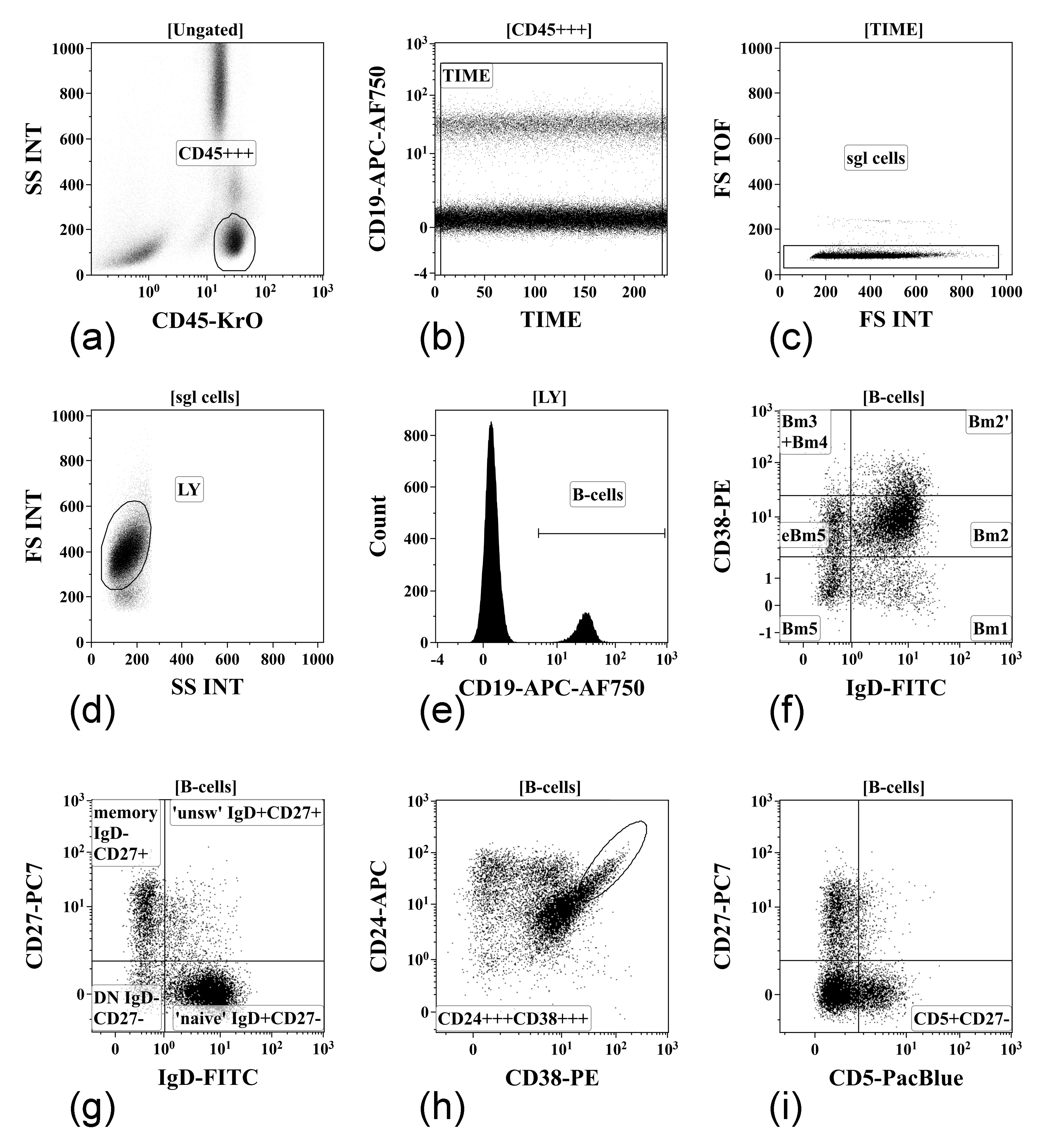


Total lymphocyte population was first gated based on their side-scatter and bright CD45 expression (dot plot A); dot plot B – artifact exclusion included time gating; then doublets were excluded from the analysis using FS-area (FS INT) and FS-height (FS TOF) on dot plot C. Next, FS vs SS gating (dot plot D) was used to discriminate lymphocytes and cell debris. B cells were then gated as CD19+ lymphocytes (dot plot E). Finally, distinct B cell subsets were identified using different patterns of co-expressing antigens. Co-expression of IgD and CD38 (so-called “Bm1-Bm5” classification, dot plot F) allowed to purify “virgin naïve” Bm1 cell IgD+CD38-, “activated naïve” Bm2 cells (IgD+CD38+), pre-germinal-center Bm2’ cells (IgD+CD38++), common subset, containing centroblasts and centrocytes (so-called “Bm3+Bm4” cells, IgD-CD38++), early memory and resting memory cells (eBm5 and Bm5 cells with the following phenotypes – IgD-CD38+ and IgD-CD38-, respectively). Next, on the dot plot G (IgD vs. CD27 expression) we distinguished between “naïve” cells (IgD+CD27–) and three types of memory cells – “unswitched” memory cells (‘unsw’ IgD+CD27+), “class-switched” memory cells ( memory IgD–CD27+) and co-called “double-negative” memory cells (DN IgD–CD27–). Transitional B cells were identified as CD24+++CD38+++ (dot plot H). Finally, CD5-expressing B cell were purified within total B cell subset on dot plot I (marked as “CD5+CD27-“).

**Supplementary Figure 2.** Follicular T helper cells flow cytometry immunophenotyping gating strategy.


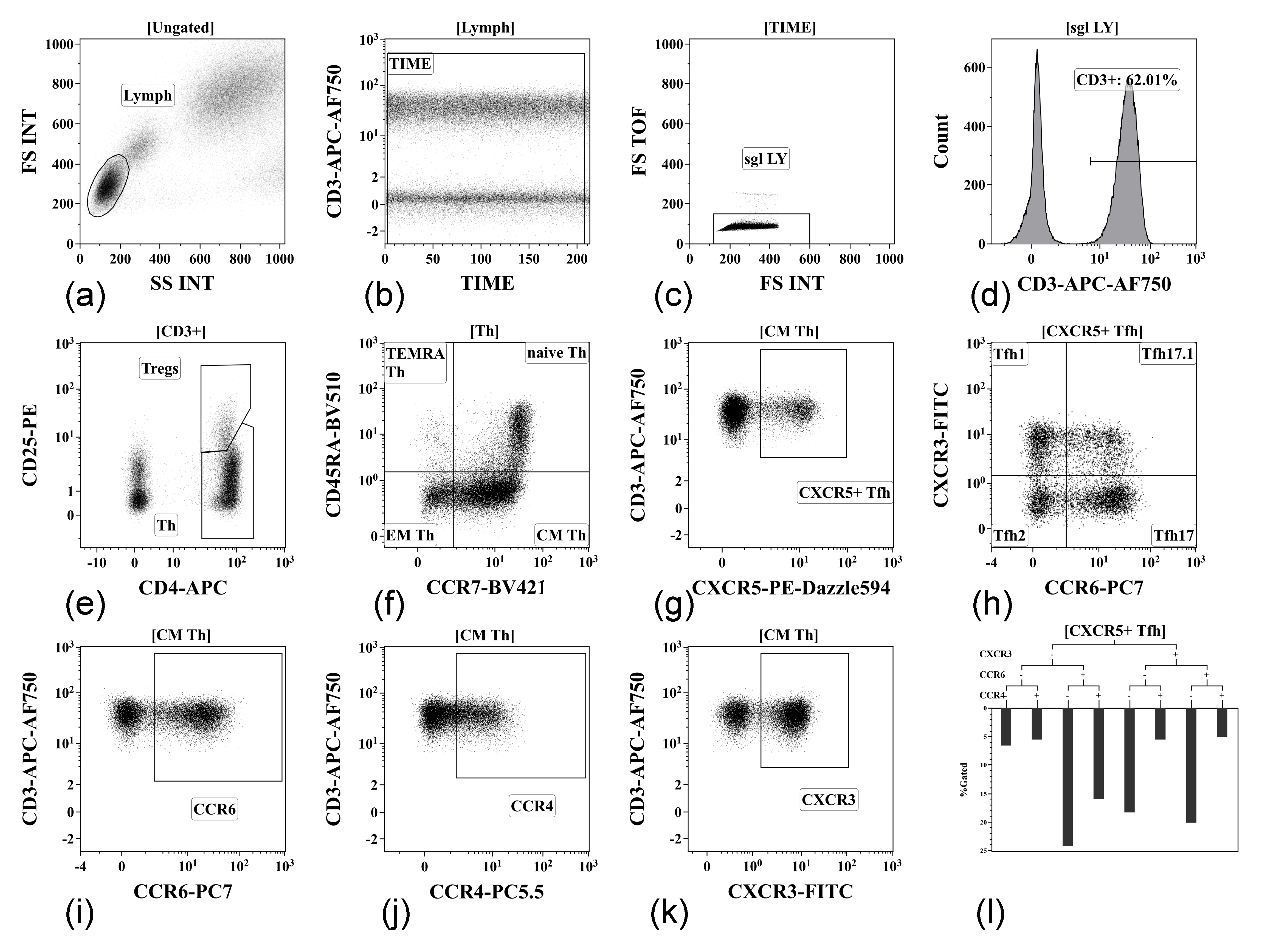


Total lymphocyte population was first gated based on their side-scatter and forward-scatter (dot plot A); dot plot B – artifact exclusion included time gating; then doublets were excluded from the analysis using FS-area (FS INT) and FS-height (FS TOF) on dot plot C. Next, based on CD3 expression (dot plot D) the total T cell subset was identified. T cells were further analyzed by expression of CD25 and CD4 (dot plot E) for identification of total regulatory T cells (“Tregs”) and conventional T helper (“Th”) cells. Within total Th cells we purified central memory subset (dot plot F, marked as “CM Th”) with CD45RA-CCR7+ phenotype that contained follicular Th cells (Tfh). Finally, based on CXCR5 expression (dot plot G) the total Tfh cell subset was identified. We used two gating strategies for circulating Tfh cell subsets by flow cytometric analysis. Primarily, we analyzed the cell-surface expression of the chemokine receptors CXCR3 and CCR6 (dot plot H) to identify CXCR3+CCR6− Tfh1-, CXCR3−CCR6− Tfh2-, CXCR3−CCR6+ Tfh17-like as well as Th17.1-like cells. Next, in order to study CCR4, CCR6 and CXCR3 co-expression on Tfh cells we used hierarchical tree histograms. Dot plots I, J and K – expression of CCR6, CCR4 and CXCR3, respectively, by CM Th. The regions “CCR6”, “CCR4” and “CXCR3” were used as branches for hierarchical tree histograms. Hierarchical tree histogram L was gated on CM CXCR5+ Tfh subset. The frequency histogram below the trees indicates the relative proportion of cells with different patterns of CCR6, CCR4 and CXCR3 co-expression within CM Tfh.

**Supplementary Figure 3. Correlations between** the percentages of CXCR5+ CM Th cells and the values of Bm1 B cell subset in sarcoidosis (a) and healthy control (b) groups.


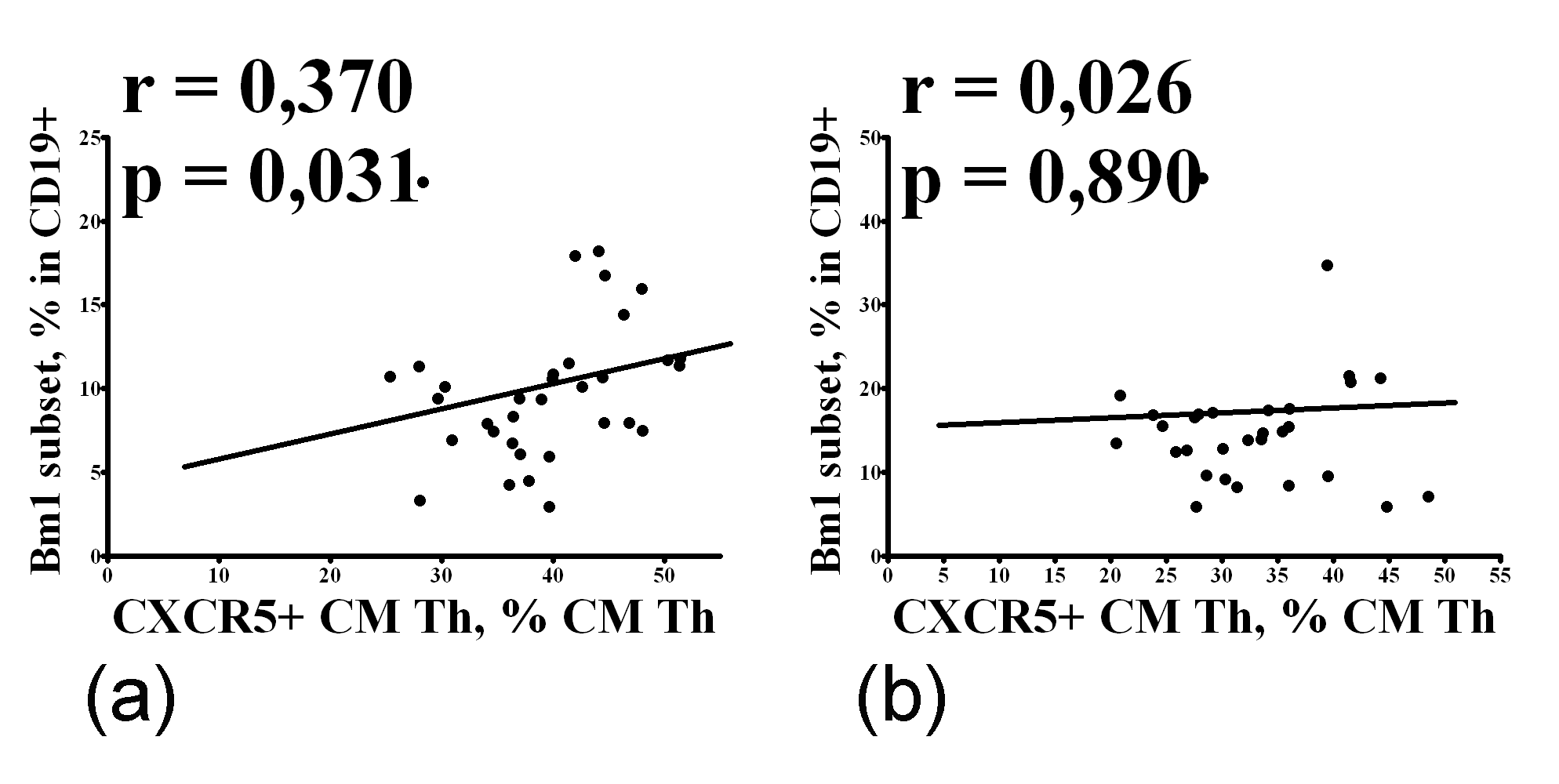


**Supplementary Table 1. Correlations between values of B cell subsets and Tfh subsets in sarcoidosis patients.**

| **Subsets** | **Tfh1** | **Tfh2** | **Tfh17** | **DP** |
| --- | --- | --- | --- | --- |
| Total CD19+ cells, % LY | -0.250  0.154 | 0.052  0.771 | 0.226  0.199 | -0.113  0.525 |
| Bm1, % CD19+ | -0.142  0.423 | -0.013  0.941 | 0.138  0.436 | 0.097  0.583 |
| Bm2, % CD19, % CD19++ | 0.186  0.292 | 0.055  0.759 | -0.188  0.288 | -0.160  0.367 |
| Bm2', % CD19+ | -0.162  0.361 | -0.068  0.702 | 0.138  0.438 | -0.023  0.896 |
| “Bm3+Bm4”, % CD19+ | 0.030  0.864 | 0.026  0.886 | -0.100  0.574 | 0.161  0.364 |
| eBm5, % CD19+ | 0.158  0.372 | -0.007  0.968 | -0.173  0.328 | 0.172  0.332 |
| Bm5, % CD19+ | 0.031  0.864 | -0.013  0.943 | -0.028  0.877 | 0.134  0.448 |
| CD24+++CD38+++, % CD19+ | **-0.387**  **0.024** | 0.044  0.803 | **0.342**  **0.048** | -0.073  0.680 |
| CD5+CD27-, % CD19+ | -0.111  0.533 | -0.212  0.229 | 0.154  0.385 | 0.116  0.515 |
| IgD-CD27+, % CD19+ | 0.095  0.592 | 0.015  0.932 | -0.132  0.458 | 0.196  0.266 |
| IgD+CD27-, % CD19+ | -0.066  0.712 | 0.042  0.815 | 0.072  0.685 | -0.202  0.252 |
| IgD+CD27+, % CD19+ | -0.010  0.956 | -0.143  0.419 | 0.064  0.717 | 0.177  0.316 |
| IgD-CD27-, % CD19+ | 0.196  0.267 | -0.089  0.618 | -0.172  0.331 | 0.135  0.446 |

**Note:** in supplementary tables 1-4 significant correlations between T- and B-cell subsets were highlighted in bold. Correlations were assessed using Spearman’s rank correlation coefficient. The differences between the groups were considered significant when p values were <0.05. In each cell of the table, the upper value corresponds to r values while the lower value corresponds to p values.

**Supplementary Table 2. Correlations between values of B cell subsets and Tfh subsets in healthy control group.**

| **Subsets** | **Tfh1** | **Tfh2** | **Tfh17** | **DP** |
| --- | --- | --- | --- | --- |
| Total CD19+ cells, % LY | -0.116  0.540 | 0.168  0.376 | 0.112  0.554 | -0.065  0.734 |
| Bm1, % CD19+ | -0.300  0.107 | 0.155  0.413 | 0.230  0.222 | 0.007  0.969 |
| Bm2, % CD19, % CD19++ | **0.435**  **0.016** | 0.154  0.417 | -0.377  0.040 | -0.281  0.132 |
| Bm2', % CD19+ | 0.357  0.053 | 0.093  0.626 | -0.270  0.149 | -0.222  0.239 |
| “Bm3+Bm4”, % CD19+ | 0.084  0.659 | 0.018  0.926 | -0.083  0.664 | -0.106  0.578 |
| eBm5, % CD19+ | -0.140  0.459 | -0.189  0.318 | 0.094  0.621 | 0.244  0.194 |
| Bm5, % CD19+ | **-0.568**  **0.001** | -0.220  0.243 | **0.505**  **0.004** | 0.349  0.059 |
| CD24+++CD38+++, % CD19+ | 0.375  0.041 | 0.109  0.566 | -0.245  0.191 | -0.293  0.116 |
| CD5+CD27-, % CD19+ | 0.017  0.930 | 0.005  0.979 | 0.082  0.666 | -0.192  0.308 |
| IgD-CD27+, % CD19+ | **-0.434**  **0.017** | -0.267  0.154 | 0.360  0.051 | **0.378**  **0.040** |
| IgD+CD27-, % CD19+ | 0.253  0.177 | -0.018  0.925 | -0.157  0.406 | -0.150  0.430 |
| IgD+CD27+, % CD19+ | -0.028  0.884 | 0.248  0.187 | -0.066  0.729 | -0.075  0.695 |
| IgD-CD27-, % CD19+ | -0.313  0.093 | -0.142  0.455 | 0.331  0.074 | 0.149  0.431 |

**Supplementary Table 3. Correlations between values of B cell subsets and Tfh subsets, purified based on co-expression of CCR6, CCR4 and CXCR3 in patients with sarcoidosis.**

| **Subsets** | CXCR3-  CCR6-  CCR4- **Tfh** | **Tfh2** | **Tfh17** | **CCR4+ Tfh17** | **Tfh1** | CXCR3+  CCR6-  CCR4+ **Tfh** | **Tfh17.1** | **DP Tfh17** |
| --- | --- | --- | --- | --- | --- | --- | --- | --- |
| Total CD19+ cells, % LY | 0.006  0.973 | 0.019  0.915 | 0.220  0.211 | -0.018  0.917 | -0.133  0.454 | -0.296  0.090 | -0.054  0.761 | -0.246  0.161 |
| Bm1, % CD19+ | 0.013  0.940 | -0.160  0.365 | 0.182  0.302 | 0.026  0.886 | -0.125  0.481 | -0.175  0.323 | 0.071  0.688 | 0.039  0.828 |
| Bm2, % CD19, % CD19+ | 0.150  0.397 | -0.023  0.896 | -0.031  0.860 | -0.234  0.183 | 0.213  0.225 | -0.050  0.778 | -0.085  0.632 | -0.312  0.072 |
| Bm2', % CD19+ | -0.158  0.371 | 0.112  0.527 | 0.006  0.975 | 0.176  0.319 | -0.097  0.584 | -0.049  0.784 | -0.061  0.732 | 0.113  0.525 |
| “Bm3+Bm4”, % CD19+ | -0.213  0.227 | 0.309  0.075 | **-0.485**  **0.004** | **0.349**  **0.043** | -0.146  0.411 | **0.518**  **0.002** | -0.014  0.937 | **0.592**  **<0.001** |
| eBm5, % CD19+ | -0.007  0.966 | -0.021  0.906 | -0.189  0.285 | -0.020  0.911 | 0.069  0.697 | 0.248  0.157 | 0.147  0.407 | 0.169  0.339 |
| Bm5, % CD19+ | -0.019  0.915 | -0.047  0.790 | -0.013  0.944 | 0.024  0.894 | -0.029  0.870 | 0.097  0.584 | 0.127  0.473 | 0.183  0.300 |
| CD24+++CD38+++, % CD19+ | -0.076  0.669 | 0.220  0.212 | 0.224  0.203 | 0.209  0.235 | -0.273  0.118 | -0.177  0.316 | -0.086  0.629 | 0.037  0.836 |
| CD5+CD27-, % CD19+ | -0.178  0.314 | -0.083  0.640 | 0.290  0.096 | -0.115  0.516 | 0.051  0.774 | -0.309  0.076 | 0.195  0.269 | -0.261  0.136 |
| IgD-CD27+, % CD19+ | 0.009  0.960 | -0.034  0.847 | -0.155  0.381 | 0.003  0.986 | 0.008  0.964 | 0.213  0.228 | 0.146  0.410 | 0.203  0.250 |
| IgD+CD27-, % CD19+ | 0.014  0.939 | 0.106  0.551 | 0.106  0.550 | -0.004  0.982 | -0.013  0.943 | -0.108  0.545 | -0.150  0.396 | -0.172  0.331 |
| IgD+CD27+, % CD19+ | 0.056  0.752 | **-0.373**  **0.030** | 0.140  0.430 | -0.105  0.556 | 0.025  0.889 | -0.245  0.163 | 0.142  0.423 | -0.059  0.740 |
| IgD-CD27-, % CD19+ | -0.117  0.508 | -0.007  0.966 | -0.119  0.503 | -0.040  0.824 | 0.125  0.481 | 0.217  0.219 | 0.171  0.335 | 0.144  0.416 |

**Supplementary Table 4. Correlations between values of B cell subsets and Tfh subsets, purified based on co-expression of CCR6, CCR4 and CXCR3 in healthy control group.**

| **Subsets** | CXCR3-  CCR6-  CCR4- Tfh | Tfh2 | Tfh17 | CCR4+ Tfh17 | Tfh1 | CXCR3+  CCR6-  CCR4+ Tfh | Tfh17.1 | DP Tfh17 |
| --- | --- | --- | --- | --- | --- | --- | --- | --- |
| Total CD19+ cells, % LY | 0.220  0.243 | 0.089  0.639 | 0.113  0.551 | -0.009  0.964 | -0.118  0.536 | -0.281  0.132 | -0.022  0.910 | -0.265  0.158 |
| Bm1, % CD19+ | 0.227  0.228 | -0.139  0.464 | **0.365**  **0.047** | -0.306  0.100 | -0.236  0.209 | **-0.461**  **0.010** | 0.064  0.738 | **-0.553**  **0.002** |
| Bm2, % CD19, % CD19+ | 0.125  0.510 | 0.212  0.261 | **-0.440**  **0.015** | 0.098  0.608 | 0.357  0.053 | **0.418**  **0.021** | -0.325  0.080 | 0.263  0.161 |
| Bm2', % CD19+ | 0.065  0.734 | 0.160  0.398 | -0.320  0.085 | -0.152  0.421 | 0.318  0.086 | 0.258  0.168 | -0.224  0.233 | 0.078  0.683 |
| “Bm3+Bm4”, % CD19+ | -0.062  0.746 | 0.283  0.130 | -0.202  0.285 | 0.146  0.441 | 0.078  0.683 | 0.261  0.164 | -0.158  0.403 | 0.054  0.776 |
| eBm5, % CD19+ | -0.137  0.471 | -0.302  0.104 | 0.183  0.334 | -0.094  0.623 | -0.092  0.628 | -0.134  0.480 | 0.278  0.136 | -0.062  0.746 |
| Bm5, % CD19+ | -0.175  0.354 | -0.264  0.158 | **0.534**  **0.002** | 0.064  0.736 | **-0.501**  **0.005** | **-0.404**  **0.027** | **0.387**  **0.035** | -0.099  0.603 |
| CD24+++CD38+++, % CD19+ | 0.072  0.704 | 0.187  0.323 | -0.305  0.102 | -0.087  0.646 | 0.330  0.075 | 0.253  0.177 | -0.285  0.126 | 0.033  0.862 |
| CD5+CD27-, % CD19+ | 0.022  0.910 | -0.012  0.949 | -0.024  0.899 | 0.116  0.540 | -0.017  0.928 | 0.147  0.437 | -0.195  0.303 | 0.178  0.347 |
| IgD-CD27+, % CD19+ | -0.233  0.215 | -0.288  0.123 | **0.410**  **0.025** | 0.030  0.873 | -0.360  0.051 | -0.317  0.088 | **0.411**  **0.024** | -0.063  0.739 |
| IgD+CD27-, % CD19+ | -0.049  0.798 | 0.123  0.519 | -0.275  0.142 | 0.184  0.330 | 0.167  0.378 | **0.374**  **0.042** | -0.192  0.311 | 0.361  0.050 |
| IgD+CD27+, % CD19+ | 0.281  0.132 | -0.026  0.890 | 0.128  0.501 | **-0.421**  **0.021** | 0.042  0.824 | -0.332  0.073 | -0.015  0.938 | **-0.539**  **0.002** |
| IgD-CD27-, % CD19+ | -0.095  0.616 | -0.180  0.341 | 0.323  0.082 | 0.015  0.938 | -0.271  0.148 | -0.248  0.185 | 0.179  0.344 | -0.144  0.448 |
